# Advances in causal discovery methods for ecological time series

**DOI:** 10.1002/brv.70180

**Published:** 2026-05-15

**Authors:** Kenta Suzuki, Masato Yamamichi, Yutaka Osada, Masayuki Ushio, Kohei Nakajima, Hiroshi Masuya, Shohei Shimizu

**Affiliations:** ^1^ Integrated Bioresource Information Division BioResource Research Center, RIKEN Tsukuba 305‐0074 Ibaraki Japan; ^2^ Institute for Multidisciplinary Sciences, Yokohama National University Yokohama 240‐8501 Kanagawa Japan; ^3^ Center for Frontier Research, National Institute of Genetics Mishima 411‐8540 Shizuoka Japan; ^4^ Genetics Program Graduate Institute for Advanced Studies, SOKENDAI (The Graduate University for Advanced Studies) Mishima 411‐8540 Shizuoka Japan; ^5^ School of the Environment, The University of Queensland Brisbane 4072 Queensland Australia; ^6^ Department of International Health and Medical Anthropology Institute of Tropical Medicine, Nagasaki University Nagasaki 852‐8523 Nagasaki Japan; ^7^ Department of Ecosystem Studies Graduate School of Agricultural and Life Sciences, The University of Tokyo Bunkyo‐ku 113‐8657 Tokyo Japan; ^8^ Advanced Institute for Marine Ecosystem Change, Tohoku University Sendai 980‐8578 Miyagi Japan; ^9^ Department of Ocean Science The Hong Kong University of Science and Technology Kowloon Hong Kong SAR; ^10^ Graduate School of Information Science and Technology, The University of Tokyo Bunkyo‐ku 113‐8656 Tokyo Japan; ^11^ Center for Advanced Intelligence Project, RIKEN Chuo‐ku 103‐0027 Tokyo Japan; ^12^ SANKEN, The University of Osaka Osaka 567‐0047 Ibaraki Japan

**Keywords:** Granger causality, transfer entropy, convergent cross mapping, empirical dynamic modelling, statistical causal inference, constraint‐based methods, functional causal models, score‐based methods, nonlinear time series analysis, ecological time series

## Abstract

Recent advances in data collection technologies (e.g. automated sensor networks, satellite remote sensing, and high‐throughput sequencing) have greatly expanded the availability of ecological time series, enabling new opportunities for causal analyses in dynamic ecosystems. Granger causality (GC) and convergent cross mapping (CCM), two prominent dynamical causal discovery methods, have gained attention in ecological studies for uncovering causal relationships in nonlinear systems. GC traces its roots to economics and was later extended through the information‐theoretic framework of transfer entropy (TE). On the other hand, CCM was developed from studies of chaotic time series. Both methods have provided critical insights into the dynamics of complex systems. In this review, we synthesize foundational concepts and recent developments in GC and CCM, exploring their respective strengths and limitations, while clarifying their interrelationship. We also review recent advances in temporal causal discovery methods, originally developed within the framework of statistical causal inference for non‐temporal data, and highlight their applicability to ecological data sets. Despite these advances, such approaches remain largely unfamiliar to ecologists. We argue that a rigorous framework of time‐series‐based causal inference, together with an appreciation of their diverse methodological developments to date, will not only raise awareness of unresolved challenges but also create new research opportunities in ecology. By offering an integrated perspective, we encourage the application and development of cutting‐edge methods in ecology to help foster a deeper understanding of ecosystem dynamics.

## I. INTRODUCTION

Temporal causal discovery, defined as the inference of causal networks (such as trophic interactions, energy flows, and communication between organisms; e.g. Brose *et al*., 2025) from multivariate time series obtained from observations and experiments, is an important starting point for understanding and managing complex ecosystems (Faust & Raes, 2012; Vacher *et al*., 2016; Toju *et al*., 2017, 2018, 2020; Röttjers & Faust, 2018; Ushio *et al*., 2018). In ecology, there is a growing expectation for the development of data‐driven methods that utilize time series, supported by advances in data collection technologies. Large‐scale time series in ecological monitoring are now increasingly available as a result of advances such as sensor networks for automated longitudinal observations, satellite remote sensing, and environmental scanning using drones (Besson *et al*., 2022). In addition, high‐throughput observations of ecological communities using advanced sequencing technologies are playing an increasingly important role (Balint *et al*., 2018), providing researchers with a powerful tool to track changes in micro‐ and macro‐organismal communities and advancing community ecology studies (e.g. Fujita *et al*., 2022; Ushio, 2022; Ushio *et al*., 2023; Yajima *et al*., 2023; Hayashi, Fujita & Toju, 2024). This paper focuses on fields that deal with the abundance of organisms and related abiotic observations, such as population ecology, community ecology, and ecosystem ecology. However, the same methodology could be extended to other areas of ecology as well, such as behavioural ecology, where it could be applied to quantitative data on movements, contacts, and other features.

### (1) Time series and causality in ecology

Ecologists have long recognized, through both field observations and experiments, that population abundances often deviate from a fixed equilibrium and can fluctuate over extended periods. Well known ecological time series include relatively regular or quasi‐regular cycles, such as predator–prey oscillations observed in laboratory microcosms (Gause, 1935) and long‐term records such as the decadal fluctuations in Canadian lynx (*Lynx canadensis*) abundance inferred from fur‐trade data (Elton & Nicholson, 1942). At the same time, other long‐running empirical studies have documented dynamics that are far less regular, including fluctuations consistent with strongly nonlinear and sometimes chaotic behaviour. Examples include laboratory insect populations in which experimental conditions generate transitions from stable dynamics to periodic and aperiodic fluctuations (Dennis *et al*., 2001), and long‐term mesocosm experiments on complex plankton communities with evidence for chaos (Beninca *et al*., 2008). In recent years, advances in monitoring technologies and the rapid growth of ecological databases have further extended this picture by making long and high‐resolution time series available across a wider range of taxa, ecosystems, and spatial scales (Clark & Luis, 2020; Rogers, Johnson & Munch, 2022).

Understanding how ecological components influence one another over time is central to many questions in ecology, from identifying trophic interactions and feedbacks to predicting ecosystem responses to environmental change (Wootton & Emmerson, 2005; Hampton *et al*., 2013). Time series of species abundances and environmental variables provide valuable records of these processes, yet causal relationships cannot be reliably inferred from simple associations in time series (e.g. correlations, synchrony, or apparent time lags) alone. This difficulty is compounded by the nonlinear dynamics, indirect effects, and feedback loops that characterize ecological systems, as well as the limited opportunities for controlled experimentation. As a result, ecologists have adopted a variety of methods, often originally developed outside of ecology, to extract causal information from observational time series. These approaches aim to infer how influences propagate through ecological systems over time, offering insights that complement experimental and mechanistic studies. Together, they form an important set of tools for addressing long‐standing ecological questions.

**Fig. 1:**
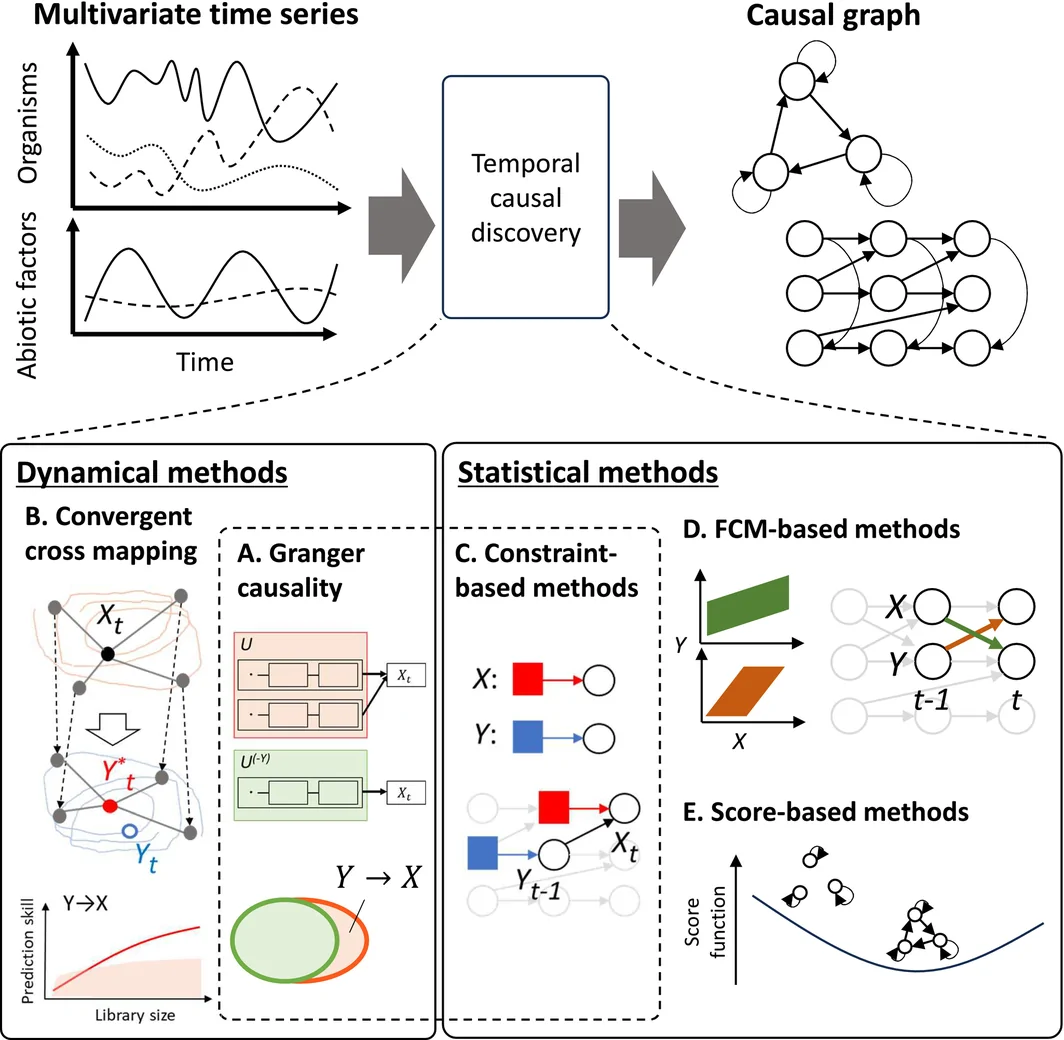
Conceptual overview of temporal causal discovery methods. Multivariate time‐series data, including organismal and abiotic variables, are analysed to infer causal graphs that capture temporal dependencies among variables. Causal discovery methods are broadly categorized into two classes: dynamical methods and statistical methods. Dynamical methods include, (A) Granger causality (GC) that detects causality based on improved prediction skill using the past values of other variables and (B) convergent cross mapping (CCM) that leverages state‐space reconstruction to identify causal influence through attractor topology and convergent prediction skill. Here, X and Y denote variables in the system, t denotes time, $X_{t}$ and $Y_{t}$ denote the states of variables X and Y at time t, U denotes the full system of variables used for prediction, and $U^{\left(-Y\right)}$ denotes the corresponding reduced system excluding *Y*. Statistical methods include, (C) constraint‐based methods that use conditional independence tests to infer the graph structure, (D) FCM (functional causal model)‐based methods that assume a structural equation model with identifiable functional relationships and (E) score‐based methods that search for the optimal causal graph by maximizing a statistical score function. These methods differ in their assumptions, theoretical foundations, and suitability for capturing ecological dynamics. Granger causality and constraint‐based methods are grouped with a dashed outline because they are closely related in that both use tests of conditional independence as a cue to constrain the causal structure. However, Granger causality is originally defined in terms of whether adding past information improves prediction; under assumptions such as linear Gaussian dynamics, it can correspond to conditional‐independence testing. At the same time, as a prediction‐based framework, Granger causality admits broader mathematical generalizations, including nonlinear, state‐space, and information‐theoretic extensions.

In this review, we distinguish between two broad classes of methods based on their primary objectives and historical development: dynamical approaches that were developed specifically to analyze temporal dynamics, and ‘statistical’ approaches that originated in statistical causal inference for non‐temporal data and were later extended to time‐series settings (Fig. 1). This distinction is made for convenience only, and it should be emphasized that dynamical approaches do not exclude the use of statistical methods.

The first objective of this review is to help both users and developers of dynamical approaches to place the associated challenges in a broader methodological context and to identify opportunities for further development. The second objective is to familiarize ecologists with recent advances in statistical methods and to facilitate their application to ecological time‐series data. In recent years, there has been a growing interest in statistical causal inference among ecologists. However, advances in the field, such as exploratory construction of causal networks and their application to time series, have yet to be fully introduced in ecology. Because concrete examples of ecological applications are still limited, this section utilizes a more enumerative approach, which may make it difficult to discern the connections among them. Nonetheless, we emphasize that there is great value in understanding the rigorous treatment of causal relationships in the field of ecological time series while remaining attentive to recent advances.

### (2) Integration of mechanistic insights

Before turning to the technical details, it is helpful to situate the approaches discussed in this review within a broader perspective on causal analysis in ecology. In ecological research, causal understanding can be supported not only by data‐driven, computational analyses, but also by mechanistic knowledge derived from theory, experiments, and prior biological understanding (Grace, 2024; Grace *et al*., 2025, 2025). By drawing on multiple lines of evidence, it becomes possible to support causal interpretations even in situations where the strict assumptions required for computational analysis are difficult to satisfy. The integrative perspective can thus ease the stringent requirements for causal identification that are often imposed as theoretical conditions. This broader view has been articulated as an integrative paradigm (Grace, 2024). From this perspective, mechanistic knowledge and mechanistic models are key complementary elements to computational causal analysis. In addition, as this review highlights, combining causal inference approaches that rely on different underlying principles may provide another important component of an integrative framework for evaluating causality in ecological systems.

Although this concise review cannot fully address this topic, it is worth briefly outlining practical ways in which mechanistic knowledge can be incorporated into causal discovery. The most direct approach is to identify, or narrow down, plausible causal links based on mechanistic understanding. For example, many physical variables (e.g. incoming solar radiation) are often treated as *exogenous drivers* at the spatial and temporal scales of an ecological study, providing strong prior constraints on plausible causal directions. Biological knowledge can also constrain candidate interactions. In planktonic predator–prey systems, prey size is not arbitrary: the sizes of prey that are typically consumed are strongly constrained by predator size, and characteristic predator–prey size ratios have been documented across major zooplankton groups (Hansen, Bjornsen & Hansen, 1994). Consequently, prey that fall far outside the feasible size range for a given predator can be ruled out as plausible trophic links. In addition to such qualitative constraints, mechanistic knowledge can also be used more formally to impose structural sparsity on model‐based causal analyses. For example, in metabolite‐mediated microbial systems, genome‐scale metabolic reconstructions and curated pathway databases can be used to pre‐specify which interactions are biochemically feasible, thereby restricting the set of non‐zero couplings to those supported by known reaction networks. In this spirit, covariance‐based approaches have used mechanistically derived stoichiometric network structure as a sparsity constraint when estimating local interaction structure from high‐dimensional metabolomics data (Sun & Weckwerth, 2012; Weckwerth, 2020).

A different strategy, distinct from using such direct constraints, is to fit mechanistic models to time‐series data, such as models expressed as differential equations, and to infer causal relationships by mapping them onto interaction parameters in the fitted model. In this approach, the presence or absence of causal relationships between variables corresponds to whether the estimated interaction strengths (interaction coefficients) are non‐zero. A wide range of approaches have been proposed for this purpose, including direct fitting of differential or difference equations to time series (Toni *et al*., 2009; Bucci *et al*., 2016), fitting multivariate autoregressive models that assume the system fluctuates around an equilibrium (Ives *et al*., 2003; Hampton *et al*., 2013), and fitting regression models that explicitly account for interspecific interactions (Ellner, Seifu & Smith, 2002; Fisher & Mehta, 2014; Suzuki *et al*., 2017). From a broader perspective, connecting mechanistic modelling with data‐driven causal analysis is likely to be especially valuable for strengthening causal interpretation in complex ecological systems. In this review, we focus on computational approaches to causal discovery from time series, while recognizing their role as one component within a broader integrative process of causal assessment.

### (3) Historical overview

We first review the dynamical approaches. In the 1980s and 1990s, the field of chaotic time series research, known as *nonlinear time series analysis*, developed with information theory as an important tool (Packard *et al*., 1980; Shaw, 1984; Kantz & Schreiber, 2004). In particular, transfer entropy (TE; Schreiber, 2000), which was developed as a method to identify causal relationships, has been used extensively in fields where long time series are relatively easy to obtain, and has led to diverse technical developments. Its applications include neuroscience (Hillebrand *et al*., 2016), climate science (Runge *et al*., 2015), physics (Runge *et al*., 2012; Ito & Sagawa, 2015), and computational social science (Borge‐Holthoefer *et al*., 2016). TE is a nonparametric (i.e. without model or data distribution assumptions) extension of Granger causality (GC; Granger, 1969) that has served as a key method in economics for decades. Thus, this line of research can be viewed more broadly as an attempt to detect causality in complex dynamics using the framework of GC. In contrast, convergent cross mapping (CCM; Sugihara *et al*., 2012), also rooted in nonlinear time series analysis, was proposed as an alternative to GC, and spurred ecologists' interest in a different approach to causal analysis of time series.

Building on prior taxonomies in the literature (Assaad, Devijver & Gaussier, 2022b; Gong *et al*., 2024; Runge *et al*., 2023), we classify statistical approaches into three families in this review, with distinct origins: constraint‐based, functional causal model (FCM)‐based, and score‐based methods. A useful common reference point is the structural equation model (SEM), which represents causal hypotheses as a set of structural equations in which each variable is expressed as a function of its direct causes plus an error term, together with a directed graph that encodes those causal dependencies (Pearl, 2000). In terms of SEMs, these families can be understood as complementary ways of specifying or learning the system of structural equations and its associated directed graph. Constraint‐based methods leverage the conditional‐independence relations implied by an underlying SEM to narrow down the set of admissible causal graphs, typically identifying an equivalence class rather than a unique structure. FCM‐based methods, in contrast, treat an SEM explicitly as a set of functional assignments (each variable as a function of its direct causes plus an error term) and seek causal directions by imposing additional assumptions on the functional form or noise. Score‐based methods pose SEM learning as a model‐selection problem, searching over candidate graphs and selecting those that optimize a score reflecting fit and complexity.

Constraint‐based methods emerged from developments in graph theory and statistics, using conditional independence tests to infer causal structure (Spirtes, Glymour & Scheines, 2000; Verma & Pearl, 2022). In parallel, FCM‐based methods have their conceptual roots in Sewall Wright's path analysis in genetics (Wright, 1921), which modelled variables as deterministic functions of their causes with the addition of independent noise terms (Denis & Legerski, 2006). This idea was later extended and formalized in econometrics as simultaneous equation models with linear, parametric assumptions (Goldberger, 1972). Around the same time, score‐based methods were developed, particularly in the context of Bayesian networks, where causal graphs are selected by optimizing a score function such as Bayesian information criteria (BIC) or Bayesian marginal likelihood (Heckerman, Geiger & Chickering, 1995). These methods were later unified under the framework of structural causal models, which formalized the connections among functional equations, graphical models, and probabilistic reasoning, providing a coherent basis for causal inference (Pearl, 2000).

In the following sections, we first review the basic concepts of the two dynamical methods, GC and CCM (Fig. 1A,B), summarize their characteristics with the latest findings, and then examine their relationship. The goal is to provide information that will enable ecologists to understand the differences between the two methods and to use them appropriately. Information theory, essential for a comprehensive understanding, is presented in Section II.5. Next, we provide an overview of the statistical methods (Fig. 1C–E) and explain how they have been developed thus far.

This review provides an overview of both dynamical and statistical methods from the perspective of their use in ecology. This contrasts with reviews such as Assaad *et al*. (2022b) and Gong *et al*. (2024), which survey a wide range of causal discovery methods for time series, without emphasizing ecological applications. Other reviews have a narrower focus, such as Runge *et al*. (2023), which highlights statistical methods, while Yuan & Shou (2022) emphasize dynamical methods. Moraffah *et al*. (2021) partially covers both dynamical and statistical methods. Although Runge (2018) is not a comprehensive review of methods, it provides a well organized overview of the fundamental issues that temporal causal inference methods need to address. Our review attempts to integrate these perspectives explicitly within ecological contexts.

## II. GRANGER CAUSALITY (GC)

Regarding causality in time series, Granger (1969) traced the origin of his formulation to a suggestion by Wiener (1956) and then proposed a more general definition: ‘We say that *Y*_t_ is causing *X*_t_ if we are better able to predict *X*_t_ using all available information than if the information apart from *Y*_t_ had been used’ (Granger, 1969, pp. 428). This definition of causality is now referred to as Granger causality (Hlaváčková‐Schindler *et al*., 2007; Amblard & Michel, 2012; Eichler, 2013; Shojaie & Fox, 2022), which can be broken down into two principles: *temporal precedence* and *uniqueness*. The principle of temporal precedence states that the cause must always precede the effect, while the principle of uniqueness states that when a variable Y affects another variable X, Y can have unique information that other variables do not have in determining the state of X.

### (1) Concept of Granger causality

A formal definition of GC incorporates a system U, which is a set of all variables in the universe according to Granger (1969). When assessing the causal relationship between X and Y, *U* necessarily includes both variables. The causal effect of variable Y on X is then obtained by comparing the predictive accuracy when using the full system U versus the reduced system $U^{\left(-Y\right)}$, that is, the system U without Y. This is an operational definition of the unique contribution of Y to the prediction of X. In practice, however, it is not realistic to assume that U contains the entire set of variables in the universe. Indeed, it is almost always a subset of the variables pertaining to the dynamical system under consideration (Eichler, 2013; Shojaie & Fox, 2022). In Section II.3, we explain the effects of unobserved confounders (variables that jointly affect *X* and *Y*) and instantaneous effects. When these factors are taken into account, GC needs to be distinguished from true causality (Eichler, 2013; Runge, 2018).

### (2) Implementation of Granger causality

**Fig. 2:**
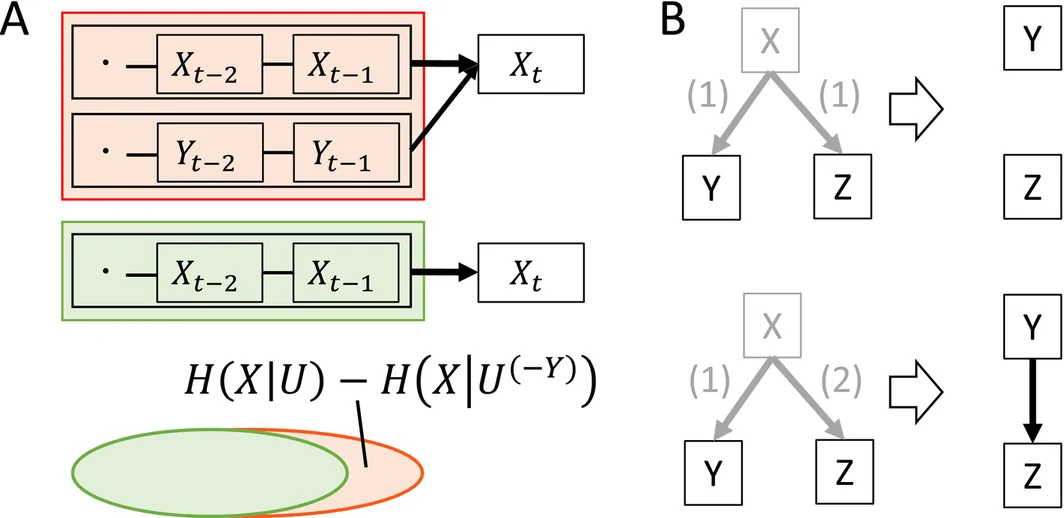
Illustration of TE calculation. (A) The sets U and $U^{\left(-Y\right)}$ in the bivariate case. TE quantifies the information flow from Y to X by taking the difference in conditional entropy computed from these two sets. Here, X and Y denote variables in the system, t denotes time, $X_{t}$ and $Y_{t}$ denote the states of variables X and Y at time t, U denotes the full system of variables used for prediction. The red elements denote prediction using the full system U, whereas the green elements denote prediction using the reduced system $U^{\left(-Y\right)}$, that is, the system excluding Y. (B) In GC, when a latent variable affects both Y and Z with no difference in time delay, no spurious causality appears between them. However, if the latent influence has different time delays, an apparent causal relationship may arise from the variable affected earlier to the one affected later. The numerical labels on the directed edges indicate the number of time steps by which the influence of X is delayed.

The definition of GC can be expressed concisely using the concepts of information theory (see Section II.5) (Barnett, Barrett & Seth, 2009) as reviewed by Hlaváčková‐Schindler *et al*. (2007) and Amblard & Michel (2012). Suppose that the state of variable X at time t is represented by $X_{t}$ and the state of the system including X before t is represented by U. Using the definition of entropy for discrete variables (see Section II.5), the uncertainty about $X_{t}$ and the remaining uncertainty in $X_{t}$ given U can be written as the Shannon entropy $H\left(X_{t}\right)=\mathrm{\Sigma P}\left(x_{t}\right)\log P\left(x_{t}\right)$ and the conditional entropy $H\left(X_{t}|U\right)=\mathrm{\Sigma P}\left(x_{t},u\right)\log P\left(x_{t}|u\right)$, respectively. Here, $P\left(x_{t}\right)$ denotes the probability that $X_{t}=x_{t}$, u denotes a realization of U, and $P\left(x_{t}|u\right)$ denotes the conditional probability that $X_{t}=x_{t}$ given U=u. Σ denotes the sum over all possible states that $X_{t}$ and U can take. Thus, the predictive accuracy when U is known is $H\left(X_{t}\right)-H\left(X_{t}|U\;\right)$. It is the uncertainty of X that U reduces. Similarly, using $U^{\left(-Y\right)}$, the above relationship can be written as $H\left(X_{t}\;\right)-H\left(X_{t}|U^{\left(-Y\right)}\right)$. Therefore, the goodness of the prediction that increases by knowing Y is:

(1) $$H\left(X_{t}|U\right)-H\left(X_{t}|U^{\left(-Y\right)}\right).$$

when this value is not zero, we say Y Granger causes X ($Y\overset{\mathrm{GC}}{\to }X$) (Fig. 2A). Comparing this to Equation (7), we see that Equation (1) is consistent with the definition of TE when $U=\left\{X,Y\right\}$. This definition is based solely on the conditional entropies of X and Y, and does not depend on how they are modeled, for example, whether they follow a stochastic process such as a Gaussian distribution or a deterministic relationship described by difference equations. In other words, TE is a non‐parametric implementation of GC in the bivariate case, and the original definition of GC encompasses its multivariate form.

There are two important points to consider when calculating GC in practice. The first is the evaluation of predictive accuracy. Since Granger (1969) used a multivariate vector autoregressive model (MVAR) for its implementation, problems arising from MVARs – such as the treatment of non‐linearity, non‐stationarity, and assumptions of Gaussian noise – are sometimes referred to as the problems of GC itself (and there is even a misunderstanding that GC was defined in a bivariate setting). However, as we have seen, the definition of GC is more general and model‐independent, and modern nonparametric implementations can, to some extent, relax these restrictive assumptions, although they may still rely on local stationarity. GC tests based on MVAR have a long history in fields such as economics (Eichler, 2013; Shojaie & Fox, 2022), and there are also studies that have shown the equivalence of MVAR‐based GC and TE for Gaussian variables (Barnett *et al*., 2009). However, for ecological dynamics in which non‐linearity and non‐stationarity play an important role, the relevance of parametric approaches with linear models is limited compared to non‐parametric methods such as TE (but see, e.g. Ives, Abbott & Ziebarth, 2010; Holmes, Ward & Kellie, 2012).

Calculating TE in a system with many variables will require an impractical amount of data (Schreiber, 2000; Runge *et al*., 2012; Novelli & Lizier, 2021). One of the key challenges then is how to determine U in its multivariate extension. Several strategies have been proposed – for example, combining nonlinear time series analysis *via* non‐uniform embedding with progressive variable selection (Vlachos & Kugiumtzis, 2010; Faes, Nollo & Porta, 2011; Novelli *et al*., 2019) or incorporating graphical modelling approaches (Runge *et al*., 2012). Nevertheless, even with these improvements, accurate estimation in multivariate systems typically demands long time series, often several hundred points or more. For example, TE (Schreiber, 2000) and its multivariate extension (decomposed transfer entropy; Runge *et al*., 2012) were tested using time series of at least 1000 points for bivariate and tetravariate settings, respectively (see online Supporting Information Table S1). Moreover, Novelli & Lizier (2021) showed that performance on a 76‐variable data set improved markedly as the time‐series length increased from 3000 to 30,000 points both in bivariate and multivariate TE (Novelli *et al*., 2019). This data requirement has hindered the widespread application of multivariate TE in ecology.

Time series variables are often observed as continuous values. The entropy of a continuous variable X can be written as follows:

(2) $$H\left(X\right)=-\int p\left(x\right)\log_{2}p\left(x\right)\mathrm{dx}.$$

in this formula, $p\left(x\right)$ is the probability density function, and thus $H\left(X\right)$ cannot be calculated directly unless it is known *a priori* or there are sufficient numbers of observations to determine the distribution. Therefore, it is worth introducing some methods for handling continuous variables. One of the simplest methods is binning, which is to divide the range of the observed values into several regions and discretize them by assigning a symbol to each region (Cover & Thomas, 1999). However, when dealing with vectors (sequence of temporal values), the number of possible combinations increases exponentially, which can undermine the reliability of the calculation (Runge *et al*., 2012). Schreiber (2000) recommended a correlation integral method to deal with this problem. Subsequently, methods such as symbolization based on the order of magnitude of successive time series (Bandt & Pompe, 2002; Staniek & Lehnertz, 2008), the neighbourhood method (Kraskov, Stögbauer & Grassberger, 2004), and bias correction (Faes *et al*., 2011) have been proposed [for an overall review, see Hlaváčková‐Schindler *et al*. (2007) and Vejmelka & Paluš (2008)].

### (3) Known issues with Granger causality

#### (a) Issues arising from the definition

One issue based on the definition of GC is non‐separability, and the other is non‐additive causality. The problem of non‐separability is as follows (Sugihara *et al*., 2012). In a deterministic dynamical system (a system described by differential or difference equations), one variable can have complete information about all the other variables. In this case, even if the variable X, whose effect we want to determine, is removed from U, the other variables retain information about X, making it impossible to evaluate the effect of X on the other variables. This motivated them to propose CCM.

Another problem, non‐additive causality, corresponds to the case where there is an interplay between variable X and Z in the effects of these variables on Y. In such cases, the contribution evaluated by simultaneously removing variables X and Z may be greater than the sum of the contributions to the prediction of Y for each of X and Z (Williams, P. L. & Beer, R. D in preparation).

#### (b) Issues arising from observations

The issues arising from the observation include the unobserved confounders and the instantaneous effects (Runge, 2018). Suppose that true causal effects are represented by an arrow (→), and that X→Y and X→Z (Fig. 2B). In this case, X is a confounder of Y and Z. If X is not included in the data, then X is an unobserved confounder of Y and Z. In this case, even though there is no direct relationship between Y and Z, there is a possibility that Y→Z or Z→Y will be detected because of the effect of X. According to the GC formulation, if the effect of X on Y and on Z occurs with the same time lag, then *Y* and *Z* both contain the same amount of information about *X* as their own past. Therefore, if *Y* and *Z* are not directly related, there is no unique information that each of them has about the other. In this case, there is no effect of the unobserved common factor *X*. On the other hand, if there is a time lag, the causality will be directional (from the variable that is affected earlier to the variable that is affected later).

Instantaneous effects are those in which a causal effect appears between variables with the same time label, and thus without any time precedence. In such cases, the causal effect may appear simultaneously, contrary to the assumption of GC. These two issues are closely related to the development of the statistical methods, and we will discuss them in more detail later.

### (4) Recent developments in Granger causality

We have already discussed the development of GC on information theoretic backgrounds. However, there have been other ongoing attempts to expand GC focusing on the treatment of multivariate time series and non‐linearity (Shojaie & Fox, 2022; Assaad *et al*., 2022b; Gong *et al*., 2024). In recent years in particular, new methods utilizing machine learning or artificial neural networks have been proposed. These methods are nonparametric (model‐free) in that they do not assume a specific model.

The kernel method has a relatively long history as a method for nonlinear extension of GC (Ancona, Marinazzo & Stramaglia, 2004). Along this line, a method based on the theory of reproducing kernel Hilbert spaces (RKHS) which embeds data into a Hilbert space (an infinite‐dimensional generalization of Euclidean space with an inner product) and searches for nonlinear dependencies was proposed (Marinazzo, Pellicoro & Stramaglia, 2008a), which was further extended to the multivariate case (Marinazzo, Pellicoro & Stramaglia, 2008b). Sindhwani, Quang & Lozano (2013) developed a scalable method of matrix‐valued kernel learning for high‐dimensional nonlinear multivariate regression and applied it to causality evaluation. As one of the most recent developments, Ren, Li & Han (2020) proposed HSIC‐Lasso‐GC where HSIC stands for the Hilbert–Schmidt independence criterion (Yamada *et al*., 2014).

The remarkable progress in neural network architecture studies has also stimulated its application to GC. There are methods based on multilayered perceptron (MLP; Tank *et al*., 2021; Marcinkevičs & Vogt, 2021), convolutional neural network (CNN; Nauta, Bucur & Seifert, 2019), long short‐term memory (LSTM; Tank *et al*., 2021), variational autoencoder (VAE; Li, Yu & Principe, 2023b), and transformer (Kong *et al*., 2024) among others (Khanna & Tan, 2020; Xu, Huang & Yoo, 2019; Schwab, Miladinovic & Karlen, 2019; Chu *et al*., 2020; Löwe *et al*., 2022; see Gong *et al*., 2024 for a review). Reservoir computing, although computationally simpler than many deep learning models, is nonetheless highly effective for nonlinear time‐series prediction (Hart, Hook & Dawes, 2020). It has been reported that reservoir approaches show the same or higher predictive ability when compared to methods with a very large number of parameters, such as LSTM and GRU (gated recurrent unit) (Sun *et al*., 2021; Jirak *et al*., 2023; Li *et al*., 2023a; Tran, Q. H. & Nakajima, K, in preparation). This has led to attempts at calculating GC using reservoir computing (Huang, Fu & Franzke, 2020a; Duggento, Guerrisi & Toschi, 2021; Suzuki, Matsuzaki & Masuya, 2022; Wang & Fu, 2022; Li *et al*., 2024). Among them, EcohNet (Suzuki *et al*., 2022) was proposed for ecological observation data and has been benchmarked using synthetic data from the multispecies Lotka–Volterra model, as well in time‐series microbial experiments (Benincà *et al*., 2008) and long‐term observation data from a lake database (Takamura & Nakagawa, 2012; Takamura, Nakagawa & Hanazato, 2017).

### (5) Concepts of information theory

#### (a) Mathematical notation

Uppercase letters $\left(X,Y,Z\right)$ denote discrete probability variables, while lowercase letters $\left(x,y,z\right)$ refer to their specific values. The set of possible values that a random variable can take is denoted by χ (i.e. x,y∈χ). The probability that X takes a specific state x is written as $P\left(X=x\right)$. The probability that X and Y take specific states x and y at the same time, that is the joint probability, is written as $P\left(X=x,Y=y\right)$. $P\left(X=x|Y=y\right)$ is the probability that X is x given that Y is y, and is called the conditional probability. However, for practical purposes, the equivalent simpler notations, $P\left(x\right)$, $P\left(x,y\right)$, and $P\left(x|y\right)$, respectively, are used instead. When explicitly dealing with temporal variables, let X and Y be observed at each time t, their values be $X_{t}=x$ and $Y_{t}=y$, and the vectors of observations up to time t represented by $X^{\left(t\right)}$ and $Y^{\left(t\right)}$, omitting their length l for simplicity of notation.

#### (b) Shannon entropy (information entropy)

Shannon entropy is a quantitative measure of uncertainty in stochastic phenomena (Shannon, 1948). For a random variable X, Shannon entropy is:

(3) $$H\left(X\right)=-\sum_{x\in \chi }P\left(x\right)\log_{2}P\left(x\right).$$

As a common practice, we use 2 as the base of the logarithm and will omit it below.

#### (c) Joint entropy

Joint entropy is the entropy of a set of probability variables, and is written as follows:

(4) $$H\left(X,Y\right)=-\sum_{x\in \chi_{x}}\;\sum_{y\in \chi_{y}}P\left(x,y\right)\log P\left(x,y\right).$$

#### (d) Conditional entropy

When X determines Y, the uncertainty of X upon knowing Y can be calculated using conditional entropy. With the conditional probability $P\left(x|y\right)$, the probability of X=x when Y=y, it can be written as:

(5) $$H\left(X|Y\right)=-\sum_{x\in \chi_{x}}\;\sum_{y\in \chi_{y}}P\left(x,y\right)\log P\left(x|y\right).$$

In this expression, we say that *X* is conditioned on *Y*.

#### (e) Mutual information

The amount of uncertainty of X that can be reduced by knowing Y, is obtained by subtracting the uncertainty of X after knowing Y from the original uncertainty, i.e. we can obtain it as $H\left(X\right)-H\left(X|Y\right)$. This is called mutual information, and is formally defined as follows:

(6) $$\mathrm{MI}\left(X;Y\right)=H\left(X\right)-H\left(X|Y\right)=-\sum_{x\in \chi_{x}}\;\sum_{y\in \chi_{y}}P\left(x,y\right)\log\frac{P\left(x,y\right)}{P\left(x\right)P\left(y\right)}.$$

Mutual information is symmetrical, i.e. $\mathrm{MI}\left(X;Y\right)=\mathrm{MI}\left(Y;X\right)$. Therefore, the causal direction of the relationship between X and Y cannot be determined by it.

#### (f) Transfer entropy

TE quantifies the information transfer (flow of information) from one variable to another by considering the direction of time (Schreiber, 2000):

(7) $$\begin{array}{rcl} {\mathrm{TE}}_{Y\to X} & = & H\left(X_{t}|X^{\left(t-1\right)}\right)-H\left(X_{t}|X^{\left(t-1\right)},Y^{\left(t-1\right)}\right)\\ & = & -\sum_{x\in \chi_{x}}\;\sum_{y\in \chi_{y}}P\left(x,x^{\left(l\right)},y^{\left(l\right)}\right)\log\frac{P\left(x|x^{\left(l\right)}\right)}{P\left(x|x^{\left(l\right)},y^{\left(l\right)}\right)}. \end{array}$$

here, $X^{\left(t-1\right)}$ and $Y^{\left(t-1\right)}$ denote the sequences prior to time t, and are practically defined as vectors of length l, $x^{\left(l\right)}$ and $y^{\left(l\right)}$, respectively. In other words, TE quantifies the extent to which the uncertainty of $X_{t}$ is reduced by conditioning on the past of both X and Y, relative to conditioning only on the past of X. Importantly, ${\mathrm{TE}}_{Y\to X}$ is generally asymmetric (i.e. ${\mathrm{TE}}_{Y\to X}\neq {\mathrm{TE}}_{Y\to X}$) and can be regarded as a special case of conditional mutual information.

## III. CONVERGENT CROSS MAPPING (CCM)

CCM was proposed by Sugihara *et al*. (2012) as a method for detecting causality in ecological dynamics. They noted that while GC is a solution to the problem that correlation (in time series, as in other data) is not direct evidence of causality, the issue of non‐separability in dealing with ecological dynamics using GC (as explained in the Section II.3) still exists, and proposed CCM as an alternative method. We focus on CCM here, since it has become the predominant alternative to GC in ecological studies, although it is not the only approach that draws on nonlinear time series analysis for causality detection. For example, Hirata & Aihara (2010) developed a method for estimating causality based on state‐space reconstruction and recurrence plots (2D visualization of state recurrences in reconstructed phase space).

### (1) Concept of CCM

**Fig. 3:**
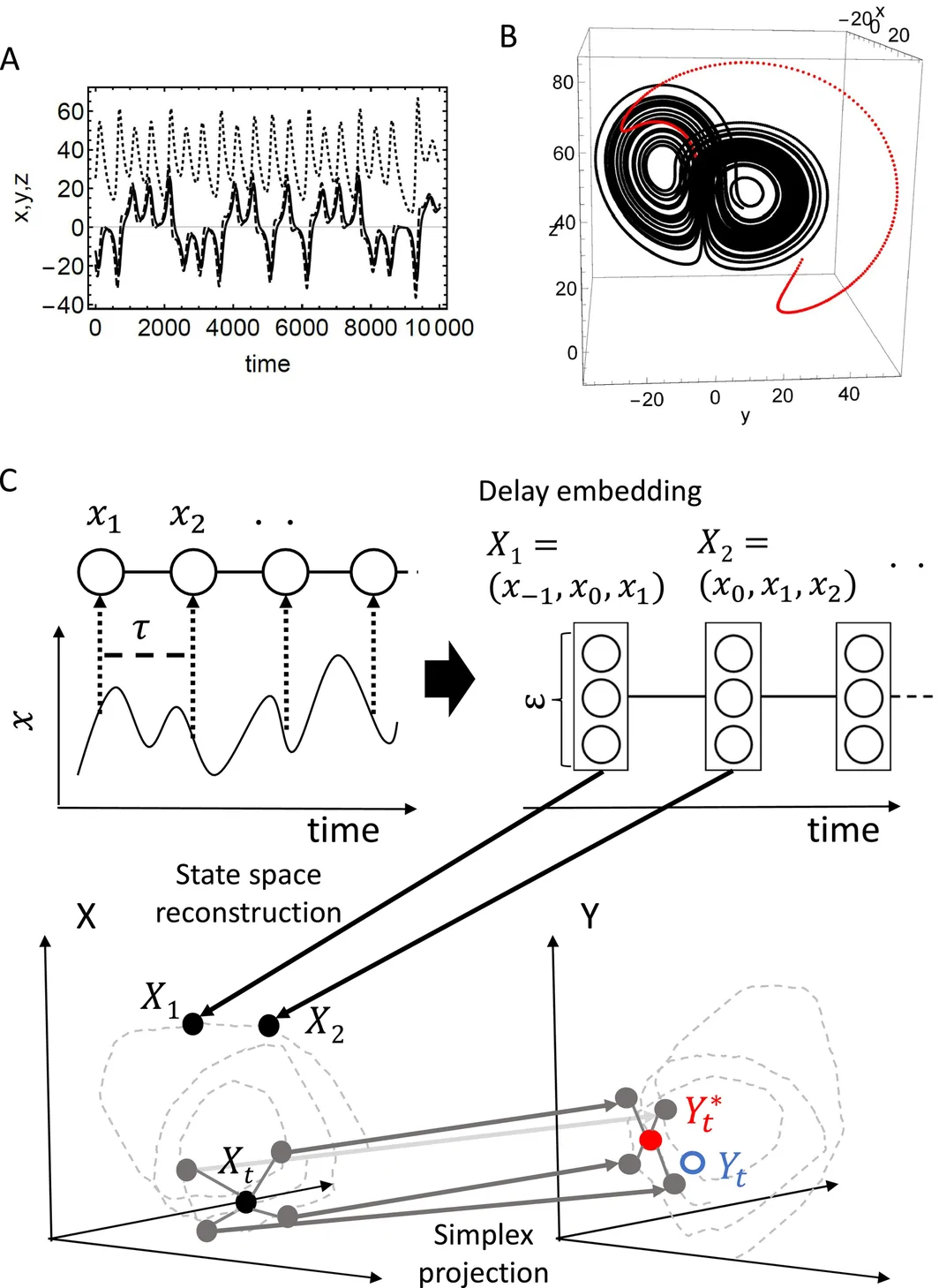
Lorenz attractor and illustration of state space reconstruction with simplex projection. (A) Time series of three variables *x*, *y*, and *z* of a Lorenz attractor. (B) The Lorenz attractor (black) and a trajectory converging toward it (red). (C) Delay embedding reconstructs the attractor in three dimensional space (ϵ=3) by taking three points at regular intervals τ from the original time series. In simplex projection, with *X* as the predictor variable and *Y* as the target variable, the nearest neighbours (grey points) of the state of *X* at time *t* ($X_{t}$) are selected. The weighted average of the same‐time projections onto *Y* provides the predicted value of *Y* at time t ($Y_{t}^{*}$; red point). The corresponding observed $Y_{t}$ is shown as a blue circle.

Deterministic dynamical systems – formulations based on differential or difference equations, are applied across biological contexts at multiple scales, from molecular diffusion dynamics to ecological processes (Murray, 2007). The path that a system follows over time in phase space, with each variable represented as an axis, is called a *trajectory*. Over time, trajectories tend to converge toward geometric structures known as *attractors*. Attractors capture the global characteristics of a system's evolution over time and take various forms: fixed points corresponding to equilibrium states, limit cycles corresponding to periodic oscillations, and more complex structures such as strange attractors with fractal geometry (e.g. the Lorenz attractor; Fig. 3A,B). Chaos, one of the characteristic behaviours of deterministic systems, arises when trajectories converge to such strange attractors. Notably, these mathematical behaviours provide a dynamical interpretation of empirical time series discussed in Section I.1.

Non‐separability can be explained under a set of conditions derived from dynamical systems theory. We assume that the state of the system evolves as a trajectory on an attractor. If the dynamics of a variable *Y* are influenced by another variable X, this is represented as a causal relationship X→Y. Here, we assume that the system is fully connected, such that any variable can be reached from any other by following these causal relationships. Under these conditions, Takens' theorem (Takens, 1980) guarantees that the geometric structure of the original attractor can be reconstructed from a single variable. The *state space reconstruction* is done using *delay embedding* (Fig. 3C), in which vectors are constructed in an ϵ‐dimensional embedding space (ϵ is called the embedding dimension) by taking ϵ points at regular intervals τ from the original time series (Kantz & Schreiber, 2004). If the above conditions hold, Takens' theorem ensures a one‐to‐one correspondence between the original attractor and its reconstruction. This correspondence implies that variables are mutually predictable by nonlinear prediction method (e.g. simplex projection; Sugihara *et al*., 2012), thereby defining them as non‐separable.

The possibility of recovering other variables from a single variable in a system where non‐separability holds implies that the single variable contains complete information about the system in which it is included. If non‐separability holds, the information that Y possesses is contained in other variables. From Equation (7), we see that if X's past contains complete information about itself, then ${\mathrm{TE}}_{Y\to X}$ becomes zero. Therefore, the definition of GC cannot be used to evaluate the causality in deterministic dynamical systems. Accordingly, Sugihara *et al*. (2012) concluded GC methods are insufficient for ecological dynamics, extending beyond purely deterministic dynamical systems, and proposed CCM as an alternative method.

### (2) Implementation of CCM

Sugihara *et al*. (2012) state that a causal relationship from *Y* to *X* ($Y\overset{\mathrm{CCM}}{\to }X$) holds when the following is satisfied for variables *X* and *Y*: (*i*) for sufficiently large *L*, the correlation coefficient between Y and $Y^{X}$, $\rho \left(Y,Y^{X}\right)$ is greater than 0; and (*ii*) $\rho \left(Y,Y^{X}\right)$ increases along with *L*, where *L* is the number of time series points used for state space reconstruction and $Y^{X}$ is Y inferred from the state space reconstruction. In addition, X and Y are required to be variables of a chaotic attractor so that ρ increases with *L*, because a chaotic attractor is composed of a dense trajectory and increasing *L* enables more accurate prediction along the trajectory. As will be explained later, the specific criteria for determining the statistical significance of conditions (*i*) and (*ii*) vary across studies.

When inferring the state of variable Y at each time point as described above, CCM uses information from both past and future states equally. In other words, the nearest neighbours of X on the reconstructed attractor are selected based on spatial rather than temporal proximity. In this sense, CCM captures causality based on the global structure of attractors (attractor topologies), unlike GC, which defines causality in terms of local and momentary temporal relationships.

In CCM, the direction of causality is determined by whether the dynamics of one attractor are embedded with another (Cummins, Gedeon & Spendlove, 2015). For example, when Y→X, the attractor reconstructed from *X* incorporates information from both *X* and *Y*, whereas the attractor for Y contains only X. Under the condition of non‐separability, even if X and Y themselves can be predicted (at least partially) from their pasts, there is asymmetry in the inference from X to Y and from Y to X (cross‐mapping). The non‐zero condition for the correlation coefficient: (*i*) is one of the criteria. Furthermore, time series obtained from field observations in particular may have unobserved confounders such as seasonality. Even under such conditions, assuming that the attractors are chaotic guarantees positive relationship (convergence) of ρ with respect to *L* only when there is causal relationship from Y to X; and (*ii*) thereby supporting the reliability of CCM as a test of the inclusion relation. This corresponds to the role of the past information in GC. In addition, the use of the surrogate method described in the next section further supports the reliability of the results.

### (3) Known issues with CCM

#### (a) Issues arising from the definition

In principle, the information contained in the reconstructed attractor is inclusive along the direction of causality. For example, consider three variables *X*, *Y*, and *Z* each following a logistic map in its temporal evolution, connected sequentially as *X* → *Y* → *Z*. In this case, the attractors reconstructed by delay embedding of *X*, *Y*, and *Z* will contain the information of *X*; *X* and *Y*; and *X*, *Y*, and *Z*, respectively. Therefore, in the relationship *X* → *Y* → *Z*, the reconstructed attractor of *Z* also contains information about *X*, meaning that the causal link *X* → *Z* cannot be ruled out. This point was noted by Sugihara *et al*. (2012), but more generally, an attractor (variable) reconstructed from a variable will contain information about all the upstream variables in the causal chain (Pecora, Caroll & Heagy, 1995; Cummins *et al*., 2015). Although this is a problem with pairwise methods in general, unless two variables are independent of the external environment (such as in an experimental system), $Y\overset{\mathrm{CCM}}{\to }X$ does not provide evidence of a direct relationship between Y and X. While it is a strength of CCM that the direction of causality between variables can be determined from the observation of a subset of variables in dynamical systems, this reliance on partial observations can be problematic when applying CCM to causal inference more generally. In order to address this issue, Ye *et al*. (2015) proposed a method for distinguishing between direct and indirect causality by evaluating CCM while shifting the time points of the variables of the source and target of inference. More recently, Leng *et al*. (2020) proposed a more straightforward approach, partial cross mapping (PCM), which uses the formula for partial correlation to distinguish between direct and indirect causality. In addition, Osada, Y., Ushio, M. & Kondoh, M. (in preparation) proposed a conditional test for distinguishing indirect effects through an information‐theoretic reformulation of CCM [Equation (10)].

#### (b) Issues arising from the implementation

When applying CCM to a given data set, several methodological decisions need to be made, such as how to determine the embedding dimension (ϵ), and how to assess if ρ is greater than zero and if it increases with L. In CCM calculations, the ϵ is often determined using the one‐step‐ahead prediction of simplex‐projection, but in nonlinear time series analysis, the false neighbour method (Kennel, Brown & Abarbanel, 1992; Cao, 1997) is more widely used (Mønster *et al*., 2017; Ye *et al*., 2019). There are two methods for determining the significance of ρ: the bootstrap method (Clark *et al*., 2015) and the surrogate method. The surrogate method includes methods that preserve the power spectrum of the time series (Ebisuzaki, 1997; Sugihara *et al*., 2012), those that preserve attractor structure (Thiel *et al*., 2006; Ushio *et al*., 2018), and those that preserve seasonality (Deyle *et al*., 2016a; Matsuzaki *et al*., 2018), and the choice among them depends on the characteristics of the time series. In addition, when determining whether the correlation coefficient increases with L, some studies use the correlation coefficient itself (Chang *et al*., 2020), while others test the difference in mean values between the two distributions (Clark *et al*., 2015). Finally, the sampling interval (τ) is usually taken from the observed time interval, but strictly speaking, it should be resampled to reduce autocorrelation (Kantz & Schreiber, 2004; Sugihara *et al*., 2012).

Sugihara *et al*. (2012) used the simplex projection as a method of inference, and subsequent studies have followed this approach, but it is only one of several available methods. For example, the CMS (cross map smoothness) approach proposed by Ma, Aihara & Chen (2014) uses a radial basis function network. More broadly, in nonlinear time series analyses, the method most suitable for prediction depends on the system dynamics (Judd & Mees, 1995). In fact, when CCM is applied to a coupled logistic map, it may fail in some parameter regions because the dynamics change discontinuously in the parameter space (Mønster *et al*., 2017).

The results of CCM are often not stable with respect to the details of implementation. This can leave room for the selection of results that are open to interpretation. If stability in this sense is not evaluated, the reliability of the results cannot be guaranteed. As CCM is a relatively recent development compared to GC, it is important to note that its implementation has not been thoroughly investigated. In this respect, both the choice of nonlinear prediction method (Judd & Mees, 1995) and the methodological insights from GC (TE), such as methods for calculating mutual information (Kraskov *et al*., 2004) and variable selection in embedding (Vlachos & Kugiumtzis, 2010; Faes *et al*., 2011; Runge *et al*., 2012), are likely to contribute to the further refinement of CCM.

### (4) Recent developments in CCM

Some of the recent developments in CCM are summarized as part of the EDM (empirical dynamic modelling; Munch, Rogers & Sugihara, 2023) framework. While CCM deals with causal relationships, there are attempts to estimate sparse ecological interactions using the related nonlinear time series method, S‐map (Suzuki *et al*., 2017; Ushio *et al*., 2018; Cenci, Sugihara & Saavedra, 2019; Chang *et al*., 2021). In addition, machine learning‐based implementations have been proposed, such as latent convergent cross mapping based on neural ordinary differential equations (ODEs) (De Brouwer *et al*., 2020) and Bayesian CCM based on deep Gaussian processes (Feng, Quirk & Djurić, 2019). Recently, reservoir computing has also been proposed as a framework for CCM‐inspired causal inference (Cao *et al*., 2025). There have also been extensions that broaden the range of applications and interpretations, such as spectrum CCM (Kitayama, Ushio & Aiba, 2021), which targets the Fourier components of the observation data, and gCCM (Gao *et al*., 2023), which focuses on spatial data and performs spatial cross‐sectional data‐based cross‐mapping prediction in the reconstructed state space.

In CCM, the correlation coefficient between time series X and Y is used as an indicator of predictive performance. There is also a method that uses mutual information as an alternative (Shi, Chen & Aihara, 2022). In this case, the difficulty of calculating entropy is a concern, but Ge & Lin (2023) overcame this by using symbolic TE (Staniek & Lehnertz, 2008). When the correlation coefficient ρ is replaced by mutual information, $\mathrm{MI}\left(X,X^{Y}\right)$ represents how much information Y has about X, and $\mathrm{MI}\left(Y^{X},Y\right)$ represents how much information X has about Y. If $\mathrm{MI}\left(X,X^{Y}\;\right)\neq \mathrm{MI}\left(Y^{X},Y\right)$ the causality is asymmetric. In other words, CCM also evaluates causality based on the information that one variable has about another. However, in this case, causality is determined based on whether each attractor contains information about another (which is recovered by state space reconstruction). This concept is closely related to the smoothness of the mapping between the reconstructed attractors (Pecora *et al*., 1995).

Information theory has played a key role in clarifying the theoretical relationship between CCM and GC. For example, Deng *et al*. (2024) showed that a CCM that relies only on the past data is equivalent to directed information (DI; Massey, 1990) for Gaussian time series. DI is a measure of causality that was proposed before TE, and it can be obtained by summing TE over all past time points. Deng *et al*. (2024) use the assumption that $H\left(x_{t}\;|X^{\left(t-1\right)}\right)=H\left(x_{t}\right)$ and $H\left(y_{t}\;|X^{\left(t-1\right)}\right)=H\left(x_{t}\;|Y^{\left(t-1\right)}\right)$, but as a result do not address the essential difference between CCM and DI (TE). However, considering that various real‐world time series have both stochastic and deterministic components, it is important to recognize that noise can blur the difference between GC and CCM.

Osada, Y., Ushio, M. & Kondoh, M. (in preparation), on the other hand, examined the analytical relationship between TE and CCM using a modified TE that measures the information flow between specific time points, i.e. information flow from $Y_{t-p}$ to $X_{t}$:

(8) $${\mathrm{TE}}_{Y_{t-p}\to X_{t}}=H\left(X_{t},|,X^{\left(\epsilon ,\tau \right)}\right)-H\left(X_{t},|,X^{\left(\epsilon ,\tau \right)},Y_{t-p}\right).$$

in this equation, unlike Equation. (3), the relationship between individual time points is made explicit, and the temporal dynamics of X are captured in the embedding vector, $X^{\left(\epsilon ,\tau \right)}$, where ϵ denotes the embedding dimension and τ denotes the sampling interval of the time series. They also showed that causal relationship of $Y_{t-p}$ to $X_{t}$ in terms of CCM can be written as follows, as the size of the training data set approaches zero:

(9) $${\mathrm{CCM}}_{Y_{t-p}\to X_{t}}^{\mathrm{IT}}=H\left(Y_{t-p}\right)-H\left(Y_{t-p}|X_{t},X^{\left(\epsilon ,\tau \right)}\right).$$

There are two differences between TE and CCM. One is that, for the causal relationship $Y_{t-p}\to X_{t}$, TE takes into account forward‐in‐time conditioning on $X_{t}$, whereas CCM takes into account backward‐in‐time conditioning on $Y_{t-p}$. The other is that CCM does not include conditioning by $X^{\left(\epsilon ,\tau \right)}$ in the former entropy term. Osada, Y., Ushio, M. & Kondoh, M. (in preparation) considered the latter and proposed the following unified information‐theoretic causality (UIC):

(10) $${\mathrm{UIC}}_{Y\to X}=H\left(Y_{t-p},|,X^{\left(\epsilon ,\tau \right)}\right)-H\left(Y_{t-p},|,X_{t},X^{\left(\epsilon ,\tau \right)}\right).$$

furthermore, they showed that this formulation is consistent with the above definition of TE by the Bayes' rule $P\left(y_{t-p},|,x_{t},x^{\left(\epsilon ,\tau \right)}\right)P\left(x_{t},|,x^{\left(\epsilon ,\tau \right)}\right)=P\left(x_{t},|,x^{\left(\epsilon ,\tau \right)},y_{t-p}\right)P\left(y_{t-p},|,x^{\left(\epsilon ,\tau \right)}\right)$. Here, $x^{\left(\epsilon ,\tau \right)}$ denotes a realization of $X^{\left(\epsilon ,\tau \right)}$. UIC can evaluate the causal effect of $Y_{t-p}$ on $X_{t}$ while distinguishing it from the effect of $X^{\left(\epsilon ,\tau \right)}$, because it includes conditioning on $X^{\left(\epsilon ,\tau \right)}$ on the denominator side. It is also robust to noise because, unlike TE, it does not include the states of Y on the conditioning side. In summary, the authors pointed out the analytical relationships between TE, CCM, and UIC by formulating them as the causal effect of a single point in the past $Y_{t-p}$ on $X_{t}$. The main differences among these approaches are summarized in Table 2. It is important to note that this is different from the definition by Schreiber *et al*. (2000) that is based on the dyadic distinction of the past and present.

## IV. STATISTICAL METHODS (METHODS BASED ON STATISTICAL CAUSAL INFERENCE)

Unlike GC and CCM, statistical methods have been developed based on non‐temporal methods of *statistical causal discovery* (Moraffah *et al*., 2021; Assaad *et al*., 2022b; Gong *et al*., 2024; Runge *et al*., 2023). Here, causal structures targeted by different approaches can be classified into two types of graph structures: window causal graphs, which explicitly consider temporal dependencies, and summary causal graphs, which capture overall dependencies without explicitly modelling temporal lags (Section IV.1). For related terminology, see Table 1.

**Table 1:** Glossary of technical terms. Definitions are provided to complement the main text. For detailed explanations and formal definitions, please refer to sources such as Runge (2018) and Peters, Janzing & Schölkopf (2017).

Term	Definition
Additive noise model (ANM)	A causal discovery method assuming additive noise in the functional relationship.
Attractor	A set of numerical values toward which a system tends to evolve.
Autocorrelation	The correlation of a signal with a delayed copy of itself.
Bayesian information criterion (BIC)	A statistical criterion for model selection.
Bayesian network	A probabilistic graphical model representing conditional dependencies.
Causal discovery	The process of identifying causal relationships from data.
Causal graph	A directed graph representing causal relationships among variables.
Causal Markov condition	Causal Markov condition states that, in a causal graph, any variable is conditionally independent of its non‐descendants, given its parents.
Causal Markov property	A fundamental property in causal inference stating that each variable is conditionally independent of its non‐descendants given its parents.
Causal stationary	The property that the causal relationships between variables remain consistent over time, even if the statistical properties (e.g. distributions) of the variables may change.
Causal sufficiency	The assumption that all common causes (confounders) of the variables being studied are included in the analysis, ensuring that no unmeasured variables influence multiple observed variables.
Chaos	A deterministic system exhibiting sensitive dependence on initial conditions.
Conditional independence	The statistical property where two variables are independent given the value of a third variable.
Conditional mutual information (CMI)	A measure quantifying the shared information between two variables, conditioned on another variable.
Convergent cross mapping (CCM)	A method for causal inference in dynamical systems based on attractor reconstruction.
Correlation coefficient	A statistical measure of the degree to which two variables move in relation to each other.
Cross map smoothness (CMS)	A technique used in nonlinear time series analysis.
Descendants	Variables that can be reached from X by following the arrows on the graph are known as descendants of X.
Direct acyclic graph (DAG)	Direct acyclic graph is a graph consisting of nodes connected by directed edges, where no sequence of edges forms a closed loop (i.e. it has no cycles).
Directed information (DI)	A measure of causal information flow in time series.
Dynamical system	A system characterized by a function that describes its time‐dependent behaviour.
Empirical dynamic modelling (EDM)	A data‐driven approach to modeling dynamic systems.
Entropy	A measure of uncertainty or randomness in a system.
Equilibrium state	A stable state where a system tends to settle over time.
Faithfulness	The graph g and the corresponding probability P are said to be faithful to each other only if the conditional independences in P are accompanied by the Markov conditions specified by g.
False nearest neighbors (FNN)	A method used to determine the embedding dimension in phase space reconstruction.
Fast causal inference (FCI)	A method for causal discovery allowing for latent confounders.
Functional causal model (FCM)	A class of models describing causal relationships using functional dependencies.
Geographical convergent cross mapping (gCCM)	A variation of CCM used for spatial data analysis.
Granger causality (GC)	A statistical hypothesis test to determine if one time series can predict another.
Greedy equivalence search (GES)	A search algorithm for structure learning in Bayesian networks.
Hilbert–Schmidt independence criterion (HSIC)	A kernel‐based measure of statistical dependence.
Identifiability	Identifiability is the ability to uniquely determine a causal effect or causal model from the observed data and the assumptions specified (e.g. the causal graph).
Independent component analysis (ICA)	A computational method for separating mixed signals into independent sources.
Information entropy	A measure of uncertainty in a probability distribution.
Information flow	The transmission of information between variables in a system.
Instantaneous effects	Causal effects that occur within the same time step.
Joint entropy	A measure of the uncertainty in two or more random variables.
Just‐in‐time linear non‐Gaussian acyclic model (JIT‐LiNGAM)	A time‐varying causal discovery model.
Latent variable	An unobserved variable that influences observed variables.
Limit cycle	A closed trajectory in phase space representing periodic behaviour.
Linear non‐Gaussian acyclic model (LiNGAM)	A causal discovery method assuming linearity and non‐Gaussian noise.
Logistic map	A mathematical model of population dynamics exhibiting chaos.
Machine learning	A field of artificial intelligence focusing on the development of algorithms that improve with data.
Markov equivalence class	A set of DAGs that represents the same set of conditional independence relationships among variables, meaning they are statistically indistinguishable based on observed data in terms of conditional independence.
Markov property	A property in which the future state of a system depends only on its present state, not past states.
Maximal ancestral graph (MAG)	A graphical representation that accounts for unobserved confounders.
Momentum conditional independence (MCI)	A technique used in statistical causal discovery.
Multivariate vector autoregressive model (MVAR)	A statistical model used to capture the linear interdependencies among multiple time series.
Mutual Information (MI)	A measure of the shared information between two variables.
Noise‐based methods	Methods in causal discovery that rely on analyzing noise distributions.
Nonlinear dynamics	The study of systems in which outputs are not proportional to inputs.
Nonparametric method	A statistical method that does not assume a specific form for the data distribution.
Non‐separability	A condition in dynamical systems where variables contain information about each other.
Optimal causation entropy (oCE)	A method for discovering causal relationships based on entropy.
Parents	A parent of a node X is a node Y that has a directed edge pointing to X, i.e. Y→X. The set of all such nodes that directly influence X is called the parents (parent set) of X, denoted as $\mathrm{Pa}\left(X\right)$.
Partial ancestral graph (PAG)	A graph representation used in causal discovery accounting for latent confounders.
Partial cross mapping (PCM)	A method for distinguishing direct from indirect causal relationships.
Peter and Clark (PC) algorithm	A constraint‐based causal discovery algorithm.
Phase space	A mathematical space representing all possible states of a dynamical system.
Principal component analysis (PCA)	A dimensionality reduction technique.
Probabilistic graphical model	A statistical model representing variables and their conditional dependencies.
Reproducing kernel Hilbert spaces (RKHS)	A mathematical framework used in statistical learning.
Reservoir computing	A neural network framework used for nonlinear time series prediction.
Simplex projection	A nonlinear prediction method used in attractor reconstruction.
S‐map	A method for modeling ecological interactions using nonlinear time series analysis.
Sparse causal graph	A causal graph with relatively few edges.
Structural vector autoregressive fast causal inference (SVAR‐FCI)	A causal discovery method for time series based on SVAR.
Structural vector autoregressive model (SVAR)	A time series model incorporating structural relationships.
Surrogate method	A statistical approach for testing causality by generating synthetic data preserving certain properties.
Takens' theorem	A theorem stating that the dynamics of a system can be reconstructed from time‐delay embeddings.
Time delay embedding	A technique for reconstructing the state space of a dynamical system.
Time series model invariant noise output (TiMINO)	A causal discovery method based on additive noise models.
Transfer entropy (TE)	A nonparametric method for detecting directional information flow in time series.
Unified information‐theoretic causality (UIC)	A method for causal inference considering information transfer.
Variational autoencoder (VAE)	A machine learning model used for unsupervised learning of latent representations.
Vector autoregressive LiNGAM (VAR‐LiNGAM)	A causal discovery method extending LiNGAM to time series.

**Table 2:** Comparison of dynamical approaches.

	Delay embedding	Conditioning of target variable on its past	Direction of prediction and causality
TE (Schreiber, 2000)	Not applied	Applied	Same
CCM (Sugihara *et al*., 2012)	Applied	Not applied	Opposite
UIC (Osada, Y., Ushio, M. & Kondoh, M. in preparation)	Applied	Applied	Opposite

**Fig. 4:**
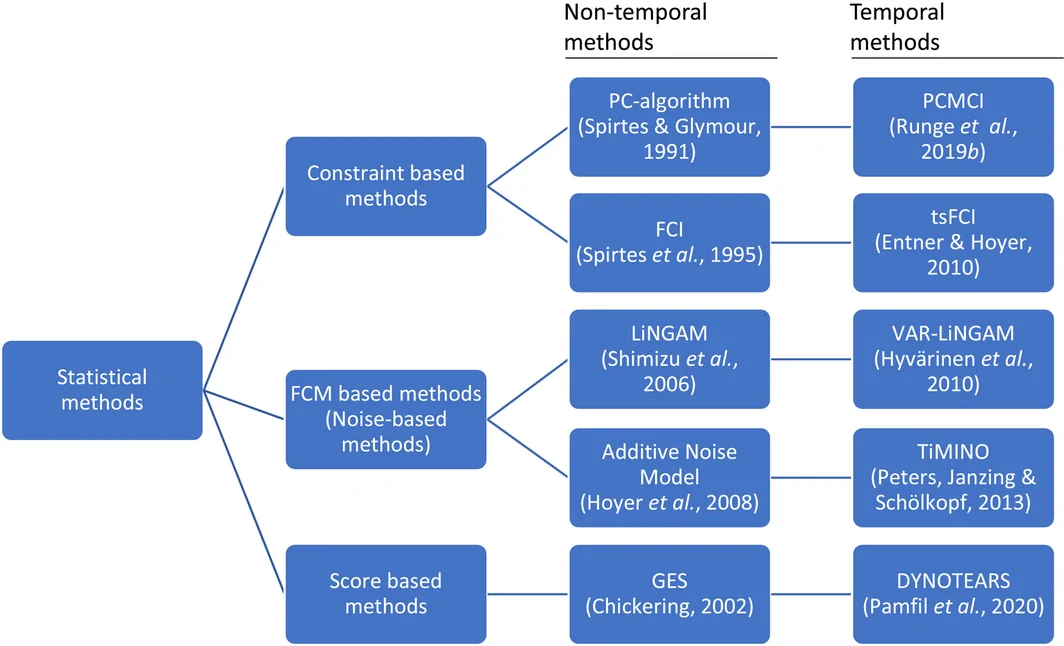
Overview of the statistical methods introduced in this section.

Statistical methods can be broadly divided into constraint‐based methods, FCM‐based methods, and score‐based methods (Fig. 4). FCM‐based methods are also called as asymmetry‐based (Runge *et al*., 2023) or noise‐based (Assaad *et al*., 2022b), depending on the focus. Figure 4 also shows the relationship between non‐temporal methods and typical temporal methods.

As already mentioned, these three families can be understood in SEM terms as complementary routes for specifying or learning a system of structural equations and the associated directed graph: FCM‐based methods posit functional assignments and leverage additional assumptions to orient causal directions, constraint‐based methods restrict admissible SEM structures using conditional‐independence constraints, and score‐based methods search over candidate graphs by optimizing a goodness‐of‐fit criterion.

**Fig. 5:**
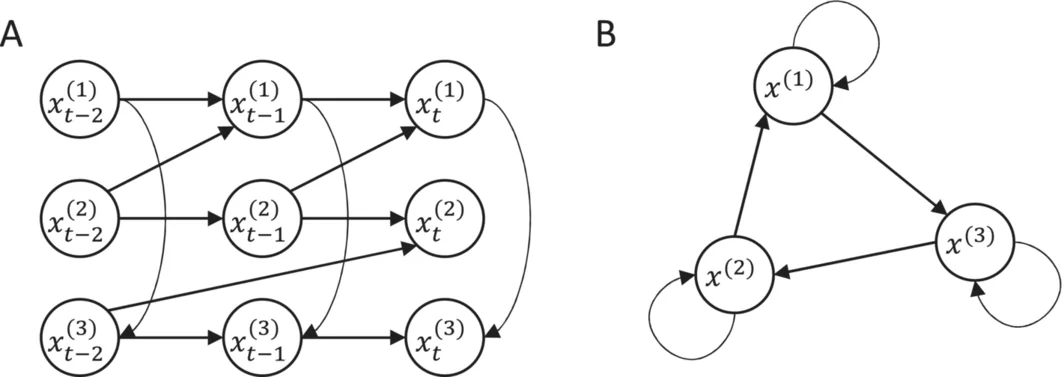
Type of causal graphs. (A) window causal graph. The link (arrow) connecting the nodes (circles) at the same time shows the instantaneous effect. This graph distinguishes instantaneous effects (links connecting nodes at the same time point) from lagged effects (links connecting nodes at different time points). (B) summary causal graph, showing the same relationship as the window causal graph, with the description of the time delay omitted.

Recent work has highlighted that SEMs can be extended to time‐series settings as dynamic structural equation models (DSEMs; Thorson *et al*., 2024), which allow specification of both instantaneous and lagged effects within a multivariate time‐series structural model (e.g. see Fig. 5A) and can accommodate missing data through efficient estimation frameworks. Ecological applications already exist in closely related SEM‐based time‐series frameworks – for example, dynamic (panel) piecewise SEMs have been used to quantify instantaneous and lagged effects in long‐term, multivariate field data sets (Farías *et al*., 2021; Almaraz, 2005). Importantly, these SEM‐based time series approaches are typically hypothesis‐driven in the sense that we need to specify a candidate causal structure *a priori* and estimate the associated path coefficients. A complementary development is the extension of directional‐separation logic to time series, where a statistical test (d‐separation test; Shipley, 2003) can be used to evaluate whether an assumed causal model is consistent with observed dependencies and to reject models that are structurally incompatible with the data (Thorson, J. T., Monnahan, C. C. & Rogers, L. A., in preparation). SEM‐based time‐series modelling provides an additional, hypothesis‐driven route for estimating and evaluating candidate causal networks.

In the reminder of this section, we will focus on constraint and FCM‐based methods, which are important for understanding the issues involved in statistical methods and their relationship with dynamical methods, but first we will look briefly at the score‐based methods. (See other reviews for more details; e.g. Assaad *et al*., 2022b; Gong *et al*., 2024). In short, score‐based methods search over the space of all possible directed acyclic graphs (DAGs: causal graphs without cyclic relationship) to maximize a goodness‐of‐fit score such as BIC. A well known algorithm for this purpose is greedy equivalence search (GES; Chickering, 2002), which iteratively searches for the optimal causal structure of a Bayesian network by adding or removing edges within equivalence classes of DAGs to maximize a scoring function. More recent studies (Zheng *et al*., 2018, 2020) reformulated the combinatorial search problem as a continuous optimization problem. In particular, Zheng *et al*. (2018) proposed NOTEARS, a continuous optimization method for learning directed acyclic graphs, and Pamfil *et al*. (2020) later extended this framework to time‐series data as DYNOTEARS (Dynamic NOTEARS).

### (1) Types of causal graphs

#### (a) Window causal graph

When explicitly considering the relationship between specific time points, the goal of statistical causal discovery for time series is to reconstruct a window causal graph. A window causal graph is a graph representation that explicitly shows the causal relationships between time points (Fig. 5A). Based on the assumption that the causal relationship is temporally invariant, causal relationships of the observed variables at each time point are shown by arrows with minimal redundancy.

#### (b) Summary causal graph

The objective of causal discovery based on the dyadic distinction between the past and present is to reconstruct the summary causal graph (Fig. 5B). Summary causal graphs are also used to show the explicit relationship between time points, i.e. those shown in a window causal graph, but excluding information on time lag (or displaying it as the annotations for arrows). In cases involving instantaneous effects, extended summary causal graphs (Assaad *et al*., 2022a) can be used.

### (2) Constraint‐based methods

Constraint‐based methods infer causal structure by using tests of conditional independence to rule out edges and thereby constrain the space of admissible graphs. In this sense, Granger causality (GC) and related approaches are closely connected to the constraint‐based family, because under commonly used assumptions, most notably linear Gaussian dynamics, GC can be expressed in terms of conditional‐independence relations. Building explicitly on the constraint‐based logic, optimal causation entropy (oCE; Sun, Taylor & Bollt, 2015) provides a theory‐driven network‐inference principle: for stationary Markov processes, the direct causal parents of a node form the minimal conditioning set that maximizes causation entropy, motivating discovery/removal procedures based on conditional mutual information (i.e. conditional‐independence) tests.

A well known algorithm for constraint‐based methods for non‐temporal data is the PC (Peter and Clark) algorithm (Spirtes & Glymour, 1991). The PC algorithm assumes causal sufficiency and causal Markov property, as well as the absence of cycles (DAG). PC momentary conditional independence (PCMCI) (Runge *et al*., 2019b) aims to reconstruct a window causal graph by determining whether there is a causal relationship between the time points of interest using the PC algorithm. After the application of the PC algorithm, it performs the following MCI (momentary conditional independence) tests to reduce false positives due to autocorrelation and improve detection power when applying the PC algorithm to time series:

(11) $$X_{t-p}⊥X_{t}|\mathrm{Pa}\left(Y_{t-p}\right),\mathrm{Pa}\left(X_{t}\right)\;Y_{t-p}.$$

here, ⊥ represents statistical independencee, p denotes the time lag, and $\mathrm{Pa}\left(X_{t}\right)$ and $\mathrm{Pa}\left(Y_{t-p}\right)$ denote the parent sets of $X_{t}$ and $Y_{t-p}$, respectively, as identified by the PC algorithm. The corresponding conditional mutual information (CMI) formulation is as follows:

(12) $$\begin{array}{l} H\left(X_{t}|\mathrm{Pa}\left(Y_{t-p}\right),\mathrm{Pa}\left(X_{t}\right)\backslash Y_{t-p}\right)\\ \;-\;H\left(X_{t}|Y_{t-p},\mathrm{Pa}\left(Y_{t-p}\right),\mathrm{Pa}\left(X_{t}\right)\backslash Y_{t-p}\right)\\ \;=0. \end{array}$$

thus, PCMCI determines the causal relationship between $Y_{t-p}$ and $X_{t}$ conditioned on both the causal parent of $Y_{t-p}$ and the parent of $X_{t},$ excluding $Y_{t-p}$. In light of the discussion so far, this formulation can be considered as a multivariate extension of TE that takes into account the flow of information between specific time points. An extension of PCMCI that can handle instantaneous effects, PCMCI+ (Runge, 2020) has been proposed.

The PC algorithm assumes causal sufficiency, whereas FCI (fast causal inference; Spirtes, Meek & Richardson, 1995; Zhang, 2008) relaxes this assumption to account for unobserved confounders. While the PC algorithm assumes a DAG, FCI assumes a maximal ancestral graph (MAG) that includes links with double‐sided arrows in order to deal with unobserved confounders. Furthermore, because it is necessary to take into account the undecidability of the link ends due to the existence of Markov equivalence classes, the FCI‐reconstructed graph structure is expressed as a partial ancestral graph (PAG), which represents such undecidability by adding circles to the link ends.

Two temporal causal discovery methods based on FCI are tsFCI (time‐series Fast Causal Inference; Entner & Hoyer, 2010) and structural vector autoregressive (SVAR)‐FCI (Malinsky & Spirtes, 2018). tsFCI reconstructs a window causal graph and can accommodate non‐linearity but is unable to account for instantaneous effects. On the other hand, SVAR‐FCI is based on a SVAR model (Swanson & Granger, 1997), which is a time‐series model based on structural causal models. By focusing only on the distinction between past and present, this method reconstructs a summary graph and can handle instantaneous effects, but not non‐linearity. LPCMCI (latent PCMCI; Gerhardus & Runge, 2020), which was recently proposed as an extension of PCMCI, is a method for reconstructing a window causal graph that can accommodate both non‐linearity and instantaneous effects.

### (3) FCM‐based methods

Constraint‐based methods can be implemented nonparametrically and can handle nonlinear relationships well, but in general they do not fully distinguish all members of a Markov equivalence class. This means that the direction of some contemporaneous (instantaneous) links may not be identifiable and can remain undirected. In contrast, the following FCM‐based method has been established as a method that theoretically guarantees the complete recovery of causal structures by making several assumptions. Linear non‐Gaussian acyclic models (LiNGAMs; Shimizu *et al*., 2006, 2011) and additive noise models (ANMs; Hoyer *et al*., 2009) are non‐temporal FCM‐based methods.

As a structural causal model, the following equations describe the causal relationship *X* → *Y* and *Y* → *X*, respectively, with Gaussian noise terms $\eta_{x}$ and $\eta_{y}$:

$$Y=a_{x}X+\eta_{x},$$

(13) $$X=a_{y}Y+\eta_{y}.$$

here, $a_{x}$ and $a_{y}$ are constants. These models are in a Markov equivalence class and thus the causal direction between X and Y is unidentifiable (Shimizu *et al*., 2006). In contrast, LiNGAM is a method that recovers causal structures, including Markov equivalence classes, by assuming a linear relationship between variables, the non‐Gaussian noise, and the DAG as the underlying causal structure. A method based on LiNGAM called vector autoregressive (VAR)‐LiNGAM (Hyvärinen, Shimizu & Hoyer, 2008; Hyvärinen *et al*., 2010) has been proposed to reconstruct window causal graphs. This method assumes that the time series is generated by the following SVAR (Swanson & Granger, 1997):

(14) $$x_{t}^{\left(i\right)}=\sum_{p=1}^{k}\sum_{j=1}^{n}b_{t-p}^{\left(j\right)}x_{t-p}^{\left(j\right)}+\eta_{t}.$$

here, k denotes the maximum time lag considered in the model, and n denotes the number of variables. As it is based on LiNGAM, VAR‐LiNGAM can account for instantaneous effects if the target system is linear and without unobserved confounders. The recently proposed just‐in‐time (JIT)‐LiNGAM (Fujiwara *et al*., 2023) is a method that applies LiNGAM to infer a local linear FCM at each time point and is applicable to time series with both non‐linearity and non‐stationarity.

On the other hand, ANMs (Hoyer *et al*., 2009) assume the following functional form:

(15) $$x_{t}^{\left(i\right)}=f\left(\mathrm{Pa}\left(x_{i}\right)\right)+\eta_{i}.$$

here, i indexes the variables in the multivariate system, and $\mathrm{Pa}\left(x_{i}\right)$ denotes the parent set of variable i. In addition to the assumption that the causal graph is a DAG, it is possible to recover Markov equivalence classes in the following cases: (*i*) f is a nonlinear function and $\eta_{i}$ is an additive Gaussian noise term; and (*ii*) f is a linear function and $\eta_{i}$ is a non‐Gaussian additive noise term (Hoyer *et al*., 2009). A method based on this finding has been proposed as time series model with independent noise (TiMINO) (Peters, Janzing & Schölkopf, 2013):

(16) $$x_{t}^{\left(i\right)}=f\left(\mathrm{Pa}\left(x_{t}^{\left(i\right)}\right),\mathrm{Pa}\left(x_{t-1}^{\left(i\right)}\right),\ldots ,\mathrm{Pa}\left(x_{t-p}^{\left(i\right)}\right),\eta_{t}^{\left(i\right)}\right).$$

here, $\mathrm{Pa}\left(x_{t}^{\left(i\right)}\right)$ denotes the parent set of variable i at time t. TiMINO can recover window causal graphs and is applicable to the nonlinear case. In addition, a method that utilizes the non‐stationarity of time series based on nonlinear independent component analysis (ICA) has also been proposed (Monti, Zhang & Hyvärinen, 2020). An FCM‐based method that can handle unobserved confounders has not been proposed so far.

### (4) Application of statistical methods for ecological time series

Constraint‐based methods have been widely studied for nonparametric implementations and their application to ecological data, where nonlinear dynamics are especially important (Turchin & Taylor, 1992; Clark & Luis, 2020; Rogers *et al*., 2022, 2023), should, at least in principle, be straightforward. Yet applications of information‐theoretic methods related to GC, including TE to ecological time series, remain somewhat limited, although there appears to be a growing recognition of their potential (Moniz *et al*., 2007; Pennekamp *et al*., 2019; Li & Convertino, 2021). FCI‐based methods (tsFCI; Entner & Hoyer, 2010; SVAR‐FCI; Malinsky & Spirtes, 2018; LPCMCI; Gerhardus & Runge, 2020) are among the best options for simultaneously addressing contemporaneous effects and capturing nonlinear dependencies. However, in addition to the differences among the methods in terms of how they handle non‐linearity and instantaneous effects, their data requirements are an important consideration as well. In general, it is rare for ecological observations to yield hundreds of time points. Indeed, both tsFCI and SVAR‐FCI are known to perform worse than GC at such sample sizes (Entner & Hoyer, 2010; Malinsky & Spirtes, 2018; see also online Supporting Information Table S1). In contrast, LPCMCI has demonstrated improved statistical power (Gerhardus & Runge, 2020; Reiser C. in preparation). However, since these validations are based on time series of 500 points (see online Supporting Information Table S1), further studies are required to establish their effectiveness under varying ecological conditions.

The FCM‐based method is particularly powerful method when instantaneous effects are strongly anticipated. However, their application to ecological data requires careful evaluation of whether the data set meets the assumptions of the methods. TiMINO and JIT‐LiNGAM are considered suitable for application to ecological data because of their ability to handle non‐linearity. It should be noted that methods that use local linearity, like JIT‐LiNGAM, have been proposed as methods for inferring interaction strength in ecology (Suzuki *et al*., 2017). Studies on non‐temporal data have shown success in handling unobserved confounders (Henao & Winther, 2011) and, more recently, in addressing cyclicality (Drton *et al*., 2025). Extending these results to time series should further facilitate their application in ecology.

## V. DISCUSSION AND FUTURE PERSPECTIVES

In this review, we first considered GC and CCM as dynamical methods for temporal causal discovery. Both GC and CCM deal with information imbalance between variables. However, in terms of time scale, while GC deals with local information imbalance, CCM deals with global information imbalance. GC exploits the fact that there is an imbalance in the information carried by variables at each point in time, and that this leads to information flow through interactions between variables. In deterministic dynamical systems, however, there is no such imbalance due to the non‐separability. Therefore, CCM exploits the imbalance of information, which reflects the inclusion relationships among attractors. However, this difference arises solely as a formal consequence of their respective definitions and should not be directly applied to the interpretation of causal relationships estimated from actual time series. As discussed below, for example, when determinism does not hold perfectly, the distinction between CCM and GC becomes more ambiguous. Further studies, including numerical experiments, are needed to investigate this point in more detail.

The introduction of CCMs is based on the idealized situation of deterministic dynamical systems. While real ecological time series undoubtedly reflect nonlinear dynamics (Turchin & Taylor, 1992; Clark & Luis, 2020; Rogers *et al*., 2022, 2023), they do not present solutions to the equations themselves. As Pennekamp *et al*. (2019) pointed out, new information arises due to stochasticity in empirical ecological time series such as demographic processes, and part of this information propagates over time. This corresponds to the analytical result that interspecific interactions mediate the flow of information between variables (Liang, 2013). Therefore, methods based on GC would also be effective for the causal discovery in ecological dynamics (Krakovská *et al*., 2018; Barraquand *et al*., 2021). The choice between CCM and GC should be guided by the dominant mode of causal transmission: CCM is preferable when focusing on a deterministic, nonlinear driver (i.e. attractor structure), whereas GC is preferable when focusing on its stochastic components. It is important to note that the stronger the deterministic component, the weaker the effectiveness of GC (Sugihara *et al*., 2012), and the stronger the stochastic component, the smaller the difference between the two (Deng *et al*., 2024).

Next, we reviewed the developments in statistical methods. Current dynamical methods used in ecology are not equipped to handle instantaneous effects and unobserved confounders in general. Ecological processes are driven primarily by the responses of organisms to the environment, and because these responses take time, true instantaneous effects are unlikely to arise from biological processes, but rather from observational artifacts. A clear example is when a system at equilibrium is perturbated by noise but sampled at intervals longer than its autocorrelation time. In such cases, since the temporal dependency between subsequent points disappears, the relationship between these variables becomes indistinguishable from a probabilistic dependency, such as covariance. As for the unobserved confounders, if there is no difference in the time lag of the effect of an unobserved confounder on multiple variables, its effect can be removed by conditioning on the history of the target variable in TE (Schreiber, 2000) and exploiting the convergence of ρ in CCM (Sugihara *et al*., 2012). Therefore, the extent to which the effects of unobserved confounders must be dealt with is related to the extent of the time delay in the transmission of causal effects in the system under consideration. The time delay of causal effects becomes important when: (*i*) the causal effects in an ecosystem with spatial sites are considered, and the propagation of the effects is parameterized by the spatial distance; (*ii*) there is a time lag in the effect of an ecological process being considered, e.g. if two predators prey on the same prey species, but the interval until the population size increases differs.

There are two points to consider when applying statistical methods to ecological time series. The first is the treatment of nonlinear deterministic dynamics (Runge, 2018; Runge *et al*., 2019a). When the sampling interval of a time series is sparse with respect to the deterministic dynamics, the linear combination of past states may be insufficient to represent the current state accurately (Deyle *et al*., 2016b). In other words, when nonlinear deterministic dynamics are important and the sampling interval is relatively sparse, a causal graph representation based on observed variables alone may be insufficient to capture the underlying causal relationships. One possible solution is to use automated observation techniques to achieve the finest possible temporal resolution, although in practice only a limited range of ecological variables can currently be monitored in this way. Another possible approach is to use nonlinear time‐series analysis, particularly embedding‐based methods combined with variable selection, such as nonuniform embedding (Vlachos & Kugiumtzis, 2010; Faes *et al*., 2011; Novelli *et al*., 2019), to obtain a more suitable representation of the relevant state variables. Although this is not straightforward, because it requires reconstructing the geometry of the underlying attractor, it may facilitate the construction of an FCM even for sparsely sampled time series. The second is the data efficiency, which is particularly important because ecological time series are often short and strongly affected by both process noise (e.g. due to demographic stochasticity) and observation noise (e.g. due to observation methods) (Ives *et al*., 2003). As we have seen, in many cases, the validation of statistical methods does not assume nonlinearity arising from deterministic dynamics, as expected in ecological time series, and the time series used are typically sufficiently long (see online Supporting Information Table S1). When adapting causality detection methods to ecological time series, it may be necessary to evaluate and take special considerations regarding this data requirement issue.

The evaluation of methods on realistic simulated time‐series data with known ground truth is important for both dynamical and statistical approaches. While these data allow direct benchmarking, the ground truth and evaluation targets must be specified carefully to avoid misleading model‐based assessments (Miki *et al*., 2025). Various choices need to be made, such as the number of species, the connectivity of the species interactions, and the functions that describe the interaction strength with respect to the population dynamics. Studies of statistical approaches can provide us with important insights here. We have seen that when the noise component is an independent Gaussian distribution, it becomes difficult to distinguish the causal direction of X and Y (Shimizu *et al*., 2006). Therefore, it should be noted that assuming a Gaussian distribution as the noise component may lead to an underestimation of the performance of the causal detection of various methods. Multiple sources of noise need to be considered in benchmarking studies. In addition, an important factor is the parameterization. Regardless of whether it is a dynamical or statistical method, there are parameters that need to be set before analysis. An arbitrary choice of parameters can often lead to a lack of fairness in benchmarking. In this regard, we can use hyperparameter selection methods in machine learning, such as Bayesian optimization (P. I. Frazier in preparation). By ensuring that parameters for each method are determined under standardized and objective criteria, automatic parameter selection can contribute to fair and unbiased methodological comparisons.

By applying different methods in parallel, we may improve our ability to infer true causal relationships from empirical time series (Marbach *et al*., 2012). Beyond parallel application, combining methods in a complementary manner represents another promising approach. As we have seen, integrating FCM‐based approach and delay embedding is one possibility as it leverages dynamical‐system structure while retaining an explicit causal interpretation. More broadly, the integrative paradigm (Grace, 2024; Grace *et al*., 2025, 2025) emphasizes that robust causal understanding can be built by combining multiple forms of evidence, not only statistical regularities but also mechanistic knowledges, which can be used to motivate, constrain, and interpret candidate causal links (e.g. by ruling out biologically implausible edges or prioritizing mechanisms that can be experimentally probed).

Causal discovery also enables quantitative assessment of the ways that influences propagate in an ecosystem, providing new insights into how community functions emerge and persist through interactions with changing environments. The continued development of causal discovery methods will therefore form an essential foundation for understanding ecological communities from a new perspective. We anticipate that the synergy between methodological innovations and advances in ecological data collection will open new horizons for causality research in ecology.

## VI. CONCLUSIONS

(1) GC and CCM capture different aspects of causality – stochastic versus deterministic – making them complementary tools for ecological time‐series analysis.
(2) Methods rooted in statistical causal inference (e.g. constraint‐based, FCM‐based, and score‐based) are increasingly relevant for ecological research, though their use remains limited among ecologists.
(3) Noise distributions, nonlinearity, non‐stationarity, and limited time series length constrain the applicability of many methods and require careful methodological choices.
(4) Fair parameter selection and benchmarking across methods are essential to avoid biased conclusions in ecological applications.
(5) The rapid growth of ecological time‐series data offers opportunities to integrate dynamical and statistical approaches, enabling more robust causal inference and fostering new ecological insights.

## Supporting information


**Table S1.** A summary of the data sets used in papers proposing representative methods.
