# Supplementary material for: Advances in causal discovery methods for ecological time series
Source: Biol Rev Camb Philos Soc. 2026 May 15;101(5):2313–36. doi: 10.1002/brv.70180 (PMC13542360; doi:10.1002/brv.70180)
Supplement: Supplementary file 1 [file BRV-101-2313-s001.docx]

| Category | Method | Reference | Data | Comparison target |
| --- | --- | --- | --- | --- |
| Dynamical | Transfer entropy (TE) | Schreiber (2000) | 100,000, 2, tent map |  |
|  |  |  | 10,000, 2, Ulam map | time delayed mutual information |
|  |  |  | 1200, 2, breath rate and instantaneous heart rate† | time delayed mutual information |
|  | Decomposed transfer entropy (DTE) | Runge *et al*. (2012) | 1000, 4, a system of stochastic delay-differential equations | mutual information |
|  |  |  | 1268, 4, daily mean sea level pressure anomalies in the winter months of 1997–2003 at four locations in Eastern Europe† | mutual information |
|  | Convergent cross mapping (CCM) | Sugihara *et al*. (2012) | [400, 1000], 2, coupled logistic map | Granger causality |
|  |  |  | 300, 5, Lotka–Volterra difference equations | Granger causality |
|  |  |  | 60, 2, experimental predator–prey system† | Granger causality |
|  |  |  | 74, 3, California current ecosystem† | Granger causality |
|  | Partial cross mapping (PCM) | Leng *et al*. (2020) | 1000, 3, Lotka–Volterra difference equations | mutual cross mapping; Granger causality; transfer entropy |
|  |  |  | 210, 20, gene expression data (DREAM4)† |  |
|  |  |  | 794, 3, plankton mesocosm† |  |
|  |  |  | 1000, 5, air pollution and cardiovascular disease data† |  |
| Statistical | Peter and Clark momentum conditional independence (PCMCI) | Runge *et al*. (2019*b*) | 468, [2, 3, 8], monthly climate index Nino (3.4 region)  and land air temperature over British Columbia† | correlation; full conditional independence |
|  |  |  | 780, 4, dependencies between monthly surface pressure anomalies for 1948–2012 in the West Pacific† | correlation |
|  |  |  | 600, 3, Heart rate–blood pressure† | mutual information |
|  |  |  | 150, [2, 5, 10, 20, 40, 60, 80, 100], random sparsely coupled and lagged nonlinear autoregressive (AR) model | correlation; full conditional independence; Peter and Clark (PC) algorithm lasso |
|  |  |  | 250, [2, 5, 10, 20, 40], random sparsely coupled and lagged nonlinear AR model | correlation; full conditional independence; PC algorithm |
|  |  |  | 500, [2, 5, 10], random sparsely coupled and lagged nonlinear AR model | mutual information; full conditional independence; PC algorithm |
|  | LPCMCI | Gerhardus & Runge (2020) | 500, [3, 5, 7, 10, 15], latent sparse linear vector autoregressive (VAR) model (lag + contemporaneous effects) | structural vector autoregressive-fast causal inference (SVAR-FCI); SVAR-really fast causal inference (SVAR-RFCI)* (Colombo *et al*., 2012) |
|  | LPCMCI | Gerhardus & Runge (2020) | NA, 3, average daily discharges of rivers in the upper Danube basin† |  |
|  | SVAR-FCI | Malinsky & Spirtes (2018) | [100, 500, 1000, 2000, 5000, 10,000], 10 (2-4), order-1 Gaussian SVAR with latent confounders |  |
|  |  |  | 330, 8, annual observations from 1967 to 1992 of various macroeconomic and political variables† |  |
|  | time-series FCI (tsFCI) | Entner & Hoyer (2010) | [100, 1000, 10,000], 2-6(1-3), randomly constructed time-series causal models with latent variables | FCI; Granger causality; group lasso; phase slope index |
|  |  |  | NA, 4, microeconomic data of growth rates of US manufacturing firms from 1973 to 2004† |  |
|  | VAR-LiNGAM (linear non-Gaussian acyclic model) | Hyvarinen *et al*. (2010) | [100, 300, 1000], 5, SVAR(1) model with non-Gaussian innovations | two-stage method; multi-channel blind deconvolution |
|  |  |  | 1200, 4, daily returns of the stock indices† |  |
|  |  |  | 54,000, 17, MEG data† |  |
|  | Just-in-time linear non-Gaussian acyclic model  JIT-LiNGAM | Fujiwara *et al*. (2023) | 20,000, 4, non-linear additive noise model |  |
|  |  |  | 20,000, 4, non-linear additive noise model with non-stationality | regression with subsequent independence test (RESIT)* (Peters *et al*., 2014); constraint-based causal discovery from heterogeneous/nonstationary data (CD-NOD)* (Huang *et al*., 2020*b*) |
|  | Time series models with independent noise (TiMINo) | Peters *et al*. (2013) | 1000, 3 [1], lagged-confounder AR model | Granger causality; time series-LiNGAM (TS-LiNGAM)* (Hyvarinen *et al*., 2010) |
|  |  |  | 2000, 4, linear-Gaussian VAR with instantaneous effects | Granger causality; TS-LiNGAM* (Hyvarinen *et al*., 2010) |
|  |  |  | 500, 3, nonlinear additive-noise AR model | Granger causality; TS-LiNGAM* (Hyvarinen *et al*., 2010) |
|  |  |  | 100–900, 2, nonlinear AR with non-additive interaction and exogenous inputs | Granger causality; TS-LiNGAM* (Hyvarinen *et al*., 2010) |
|  |  |  | 1000, 2, nonlinear AR with dependent residuals | Granger causality; TS-LiNGAM* (Hyvarinen *et al*., 2010) |
|  |  |  | 1000, 3, additive SEM with time shift | Granger causality; TS-LiNGAM* (Hyvarinen *et al*., 2010) |
|  |  |  | 296, 2, gas furnace† | Granger causality; TS-LiNGAM* (Hyvarinen *et al*., 2010) |
|  |  |  | 194, 4, Old Faithful geyser† | Granger causality; TS-LiNGAM* (Hyvarinen *et al*., 2010) |
|  |  |  | 1000, 2, abalone age and diameter† | Granger causality; TS-LiNGAM* (Hyvarinen *et al*., 2010) |
|  |  |  | 522, 2, Dairy price of butter and cheddar cheese† | Granger causality; TS-LiNGAM* (Hyvarinen *et al*., 2010) |
|  |  |  | 7265, 6, house temperature sensor data† | Granger causality; TS-LiNGAM* (Hyvarinen *et al*., 2010) |
|  | DYNOTEARS | Pamfil *et al*. (2020) | 500, 5, sparse linear SVAR with Gaussian noise | NOTEARS; lasso; LiNGAM; time-series Greedy Fast Causal Inference (tsGFCI)* (Malinsky & Spirtes 2018) |
|  |  |  | [50, 500], [5, 10, 20, 50, 100], sparse linear SVAR with Gaussian/Exponential noise | NOTEARS; lasso; LiNGAM; tsGFCI* (Malinsky & Spirtes 2018)* |
|  |  |  | 21, 100, gene expression data (DREAM4)† | dynamic Bayesian network inference based on first-order conditional dependencies (G1DBN)*; Bayesian model averaging (BMA)-based inference (ScanBMA)*; variational Bayesian state-space model (VBSSM)*; empirical Bayes dynamic Bayesian network (Ebdbnet)* (Lu *et al*., 2021) |
|  |  |  | 1257, 97, daily stock returns† | NOTEARS |

**Table S1.** A summary of the data sets used in papers proposing representative methods. ‘Data’ indicates the number of time points, the number of variables, and a brief description of the dataset. When present, latent variables are listed in square brackets. Empirical datasets are marked with †. ‘Comparison target’ denotes the methods compared on each dataset. For methods not mentioned in the main text, we provide the corresponding references and mark them with an asterisk (*). Note that “TS-LiNGAM” is not the term used in the original reference.
